# Quantitative T_1_ and T_2_ MRI signal characteristics in the human brain: different patterns of MR contrasts in normal ageing

**DOI:** 10.1007/s10334-016-0573-0

**Published:** 2016-06-22

**Authors:** Michael J. Knight, Bryony McCann, Demitra Tsivos, Elizabeth Couthard, Risto A. Kauppinen

**Affiliations:** 1 School of Experimental Psychology, University of Bristol, 12a Priory Road, Bristol, BS8 1TU UK; 2 Institute of Clinical Neuroscience, University of Bristol, Level 1 Learning and Research Building, Bristol, BS10 5NB UK; 3 North Bristol NHS Trust, Southmead Road, Westbury-on-Trym, Bristol, BS10 5NB UK; 4 Clinical Research and Imaging Centre, University of Bristol, 60 St Michael’s Hill, Bristol, BS2 8DX UK

**Keywords:** Brain, Gray matter, White matter, Contrast-to-noise ratio, T_2_ relaxation, Dephasing

## Abstract

**Objective:**

The objective of this study was to examine age-dependent changes in both T_1_-weighted and T_2_-weighted image contrasts and spin-echo T_2_ relaxation time in the human brain during healthy ageing.

**Methods:**

A total of 37 participants between the ages of 49 and 87 years old were scanned with a 3 Tesla system, using T_1_-weighted, T_2_ weighted and quantitative spin-echo T_2_ imaging. Contrast between image intensities and T_2_ values was calculated for various regions, including between individual hippocampal subfields.

**Results:**

The T_1_ contrast-to-noise (CNR) and gray:white signal intensity ratio (GWR) did not change in the hippocampus, but it declined in the cingulate cortex with age. In contrast, T_2_ CNR and GWR declined in both brain regions. T_2_ relaxation time was almost constant in gray matter and most (but not all) hippocampal subfields, but increased substantially in white matter, pointing to an age effect on water relaxation in white matter.

**Conclusions:**

Changes in T_1_ and T_2_ MR characteristics influence the appearance of brain images in later life and should be considered in image analyses of aged subjects. It is speculated that alterations at the cell biology level, with concomitant alterations to the local magnetic environment, reduce dephasing and subsequently prolong spin-echo T_2_ through reduced diffusion effects in later life.

**Electronic supplementary material:**

The online version of this article (doi:10.1007/s10334-016-0573-0) contains supplementary material, which is available to authorized users.

## Introduction

Normal ageing is associated with inevitable loss of both gray matter (GM) and white matter (WM) tissue in the brain. In addition to macroscopic morphological alterations, cerebral metabolism and haemodynamics [1], tissue and cell microstructure [2], macromolecular composition and interactions [3, 4], and chemical composition, such as water [5] and iron content [6, 7], also undergo changes with age. The deviation of one of these particular structural or functional parameters beyond its expected value for a given age range may be indicative of disease, of which the various classes of dementias are particularly relevant examples, heightening the impetus to understand brain changes in ageing. Magnetic resonance imaging (MRI) is one of the most widely employed imaging modalities to this end, owing to its non-invasive ability to provide high-resolution structural and functional images [3, 8, 9].

MRI has been widely employed for volumetric and morphometric analysis of changes in the brain [10–20]. However, brain tissue microstructure and chemistry also change with age and alter magnetic resonance parameters, subsequently influencing the contrast in various MRI modalities. However, it is not yet entirely clear what dominates the various common MR contrasts, or what physiological or chemical changes are most likely to elicit observable changes. Some work has nonetheless shown that there is likely to be substantial utility in making further investigations. Salat et al. observed a relationship between gray-to-white matter intensity ratio (GWR) and cortical thickness from T_1_-weighted images [9], and noted that this was statistically stronger than the thinning of cortical GM in ageing. The observation led to modification of a previous algorithm for the estimation of cortical thickness, making use of GWR, which led to improved discrimination between Alzheimer’s disease (AD) and control subjects [21]. It was subsequently observed [22] that decreases in T_1_-weighted image contrast were apparent in the hippocampus and limbic system as a whole in AD. Increases in GWR (decreased contrast-to-noise ratio = CNR) were also associated with decreased hippocampal volume. Given the role of the hippocampus in various diseases, there is a clear imperative to better understand how those changes can be detected in an MR image at the earliest stage.

The basis for changes in T_1_-weighted MRI signal intensities and relaxation times with age are poorly understood. Attempts have been made to explain the T_1_ in terms of iron content [7]. A correlation exists between 1/T_1_ and iron content, as deduced from analyses on post-mortem specimens, but the relationship to GWR or CNR was not sought. Similarly, decreases in magnetisation transfer (MT) both in GM and WM with age have been reported, potentially influencing T_1_ contrast. Altered MT may be due to alterations in the interactions between water and macromolecules [3, 4].

T_2_-weighted MRI is also gaining applications in high-resolution imaging for segmentation of deep GM structures, such as basal ganglia, and subsections of the hippocampal formation in aged subjects. A recent review of approaches to hippocampal subfield labelling showed that 17 of the 21 available studies use T_2_-weighted images [23]. T_2_-weighted contrast is also affected by age. Magnaldi et al. [24] first showed that T_2_-weighted contrast declined with age for various regions of the brain, including external capsule, internal capsule, corpus callosum and periventricular WM. Later, measurements of the GWR were made between WM and various subcortical structures as well as cortical GM [25]. Generally decreasing trends were found with age in T_2_-weighted images.

The ability to infer information on the properties of tissues directly from simple measures of image contrast as well as from relaxometry (and other quantitative techniques) can be seen as a way to infer its health status. Moreover, in many pathologies, microscopic or molecular changes are likely to precede macroscopic (volumetric) changes. With this in mind, an understanding of the mechanisms by which ageing determines the appearance of T_1_ and T_2_ MR images is important in their interpretation. The objective of this study was to examine tissue contrast and quantitative spin-echo T_2_ in cognitively normal, aged subjects in order to better understand the changes in MR images in different brain regions with age. We examine the contrast between individual subfields of the hippocampus and their relaxometric properties, the cingulate gyrus, caudate nucleus, and corpus callosum. The hippocampus is one of the most vulnerable brain structures in AD pathology, along with the limbic structures, including the cingulate cortex, and deep GM structures, including the caudate nucleus, also becoming affected during the progression of AD. The purpose of our analysis is to guide interpretation of MR images in a more “pathology-driven” context, aiming for a characterisation of tissue properties to complement volume and shape analyses.

## Materials and methods

### Image acquisition and participant cohort

Our participant cohort comprised a total of 37 persons (22 females, age range from 49 to 87 years, mean age 67.3 years). Participants were required to have no known psychiatric or neurological morbidities. Participants gave informed consent and ethical approval was granted by the NHS Research Ethics Committee of North Bristol-Frenchay.

All imaging was performed using a Siemens Magnetom Skyra 3 T system equipped with a 32-channel receiver head coil. The MRI protocol comprised a 3D MPRAGE and 2D multi-echo spin echo with the following parameters: MPRAGE: coronal, TR 2200 ms, TE 2.42 ms, TI 900 ms, flip angle 9°, resolution 0.34 × 0.34 × 1.60 mm^3^ (after two-fold interpolation in-plane by zero-filling in k-space), reconstructed matrix size 540 × 640 × 144 (after two-fold interpolation in-plane), acquired matrix size 152 × 320 × 144, GRAPPA factor 2 (32 integrated reference lines), time 5:25. Spin-echo: coronal, TR 4500, TE 12 ms, number of echoes 10, echo spacing 12 ms, resolution 0.34 × 0.34 × 1.7 mm^3^ (after two-fold interpolation in-plane by zero-filling in k-space, and inclusive of 15 % slice gap), reconstructed matrix size 540 × 640, 34 slices, acquired matrix size 152 × 320, 34 slices, GRAPPA factor 2 (32 integrated reference lines), time 11:07. No post-reconstruction processing was applied to alter image resolution or appearance.

### Image processing

In all scans, the manufacturer’s procedure for correction of differential coil sensitivity (the “prescan normalize”) was used to avoid, to the maximum possible extent, shading in different regions of the image that would compromise estimates of image CNR and GWR. This is performed at image reconstruction time and uses knowledge of coil sensitivity profiles.

Quantitative T_2_ maps were generated by a voxel-wise fit by a mono-exponential function in a logarithmic space after exclusion of the first echo to avoid the effects of stimulated echoes. This was performed using software written in-house. Each entire echo train was then summed to create a single T_2_-weighted image complementary to the T_2_ map. To further reduce the impact of all sources of image shading (which derive from B_1_ transmit and B_0_ inhomogeneity as well as the different receiver coil sensitivity profiles in the 32-channel array), bias field corrections were applied to the T_1_-weighted (MPRAGE) and T_2_-weighted (spin-echo) images using FSL [26]. This was not applied for fitting of T_2_ or diffusion tensor maps, nor to the resulting quantitative maps. Our available images were therefore a T_1_-weighted MPRAGE, T_2_-weighted spin-echo image, and T_2_ map. This was also reciprocated to create an R_2_ map for image registration purposes.

### Preparation and validation of hippocampal subfield masks

Masks of the various regions used were drawn manually in native space on echo-summed T_2_-weighted images. We selected subfields that could be reliably demarcated whilst providing a basis for contrast comparisons. The masks used were of the central slices of the hippocampal CA1, DG, and SL/SR/SM subfields generated from T_2_-weighted images according to the recent manual protocol [27] (as well as the total hippocampus). The motivation for choosing these temporal lobe structures is that hippocampal atrophy is a well-established finding in AD [8] and that the CA1 subfield has been reported to be affected in the early phase of the disease whereas DG is preserved [28]. The CA1 and DG are predominantly GM whereas the SL/SR/SM is predominantly WM. In our images, we were unable to distinguish between the individual stratum lacunosom (SL), stratum radiatum (SR), and stratum moleculare (SM), for which reason these three layers were collectively masked as the SL/SR/SM subfield [27]. The CA1 and DG subfields border the SL/SR/SM subfield (from opposite sides), such that comparison of the relative contrast between these two selected GM subfields with the (WM) SL/SR/SM subfield are useful comparisons. The subiculum borders the CA1, though this border is the most variable in the literature. There is no subiculum-SL/SR/SM border. The CA2 and CA3 subfields are small and difficult to reproducibly mask. The CA1, DG, and SL/SR/SM therefore fulfil the criteria of providing both GM (CA1, DG) and WM (SL/SR/SM) to contrast, being mutually adjacent and containing sufficient voxels for reliable estimates of signal intensity. A random subset of hippocampal subfield masks was re-prepared both by a second rater and by the original rater in order to determine consistency of boundary placement. Consistency was judged based on the Dice-kappa statistic and intra-class correlation [23].

### Preparation of masks of other regions

We also prepared masks of the cingulate gyrus, the WM immediately posterior to the cingulate gyrus, the caudate nucleus, globus pallidus, and corpus callosum. Masks of the cingulate gyrus were defined directly above the hippocampal body and spanned five slices. For use on T_1_-weighted images, R_2_ maps (R_2_ = 1/T_2_) were registered to T_1_-weighted space and the transformations applied to the masks originally prepared in T_2_-weighted image space. Some manual alteration was necessary since image transformations are never perfect. R_2_ maps were used for registration to the T_1_-weighted space due to the similarity in contrast between those image types. Example masks are shown in the Supplementary Information.

### Parameterising changes in signal intensity

We used two metrics of change in relative signal intensity between two regions of interest (ROI), the CNR and GWR. The CNR is given by:

$${\text{CNR}} = \frac{{\left| {E\left( {S_{\text{G}} } \right) - E\left( {S_{\text{W}} } \right)} \right|}}{{\sqrt {\sigma^{2} \left( {S_{\text{G}} } \right) + \sigma^{2} \left( {S_{\text{W}} } \right)} }}$$

with *E* the expectation (mean) and *σ* ^2^ the variance, whilst *S*_G_ and *S*_W_ contain the signal intensities in the GM and WM regions of interest, respectively. The GWR is simply:

$${\text{GWR}} = \frac{{E\left( {S_{\text{G}} } \right)}}{{E\left( {S_{\text{W}} } \right)}}$$

with the terms defined as above. Note that *S*_G_ and *S*_W_ can be quantitative T_2_ or image intensities. GWR for quantitative T_2_ images was calculated using the formula above by replacing *S*_G_ and *S*_W_ by respective T_2_ values.

In order for these formulae to represent CNR and GWR, rather than spurious aspects of imaging instrumentation, contributions to the image intensities sampled by the ROI must contain negligible contributions arising from B_0_ and B_1_ inhomogeneity, from parallel imaging reconstruction routines, and in multi-slice 2D imaging from slice profile imperfections (by using the inner slices only where these are equilibrated). The mean and variance terms are otherwise corrupted by contributions that cause CNR and GWR to represent non-local instrument instability, rather than anything of physiological origin or interest. This may be accomplished by ensuring that ROIs are anatomically close and smaller than the size over which one may anticipate the effects of magnetic or radiofrequency field inhomogeneity to be manifest. ROIs must be representative of signal variance arising due to random sources of noise, rather than representative of anatomical shape. This has motivated our choice of ROI. More is said of this in the discussion section.

Statistical analysis was performed using Matlab R2013b. Linear fits for all figures presented in the results were performed, with confidence intervals for fitted parameters and function prediction intervals computed by 500 bootstrap simulations. P-values were also computed. A table of statistics and linear fit parameters is provided in the Supplementary Information.

## Results

**Fig. 1:**
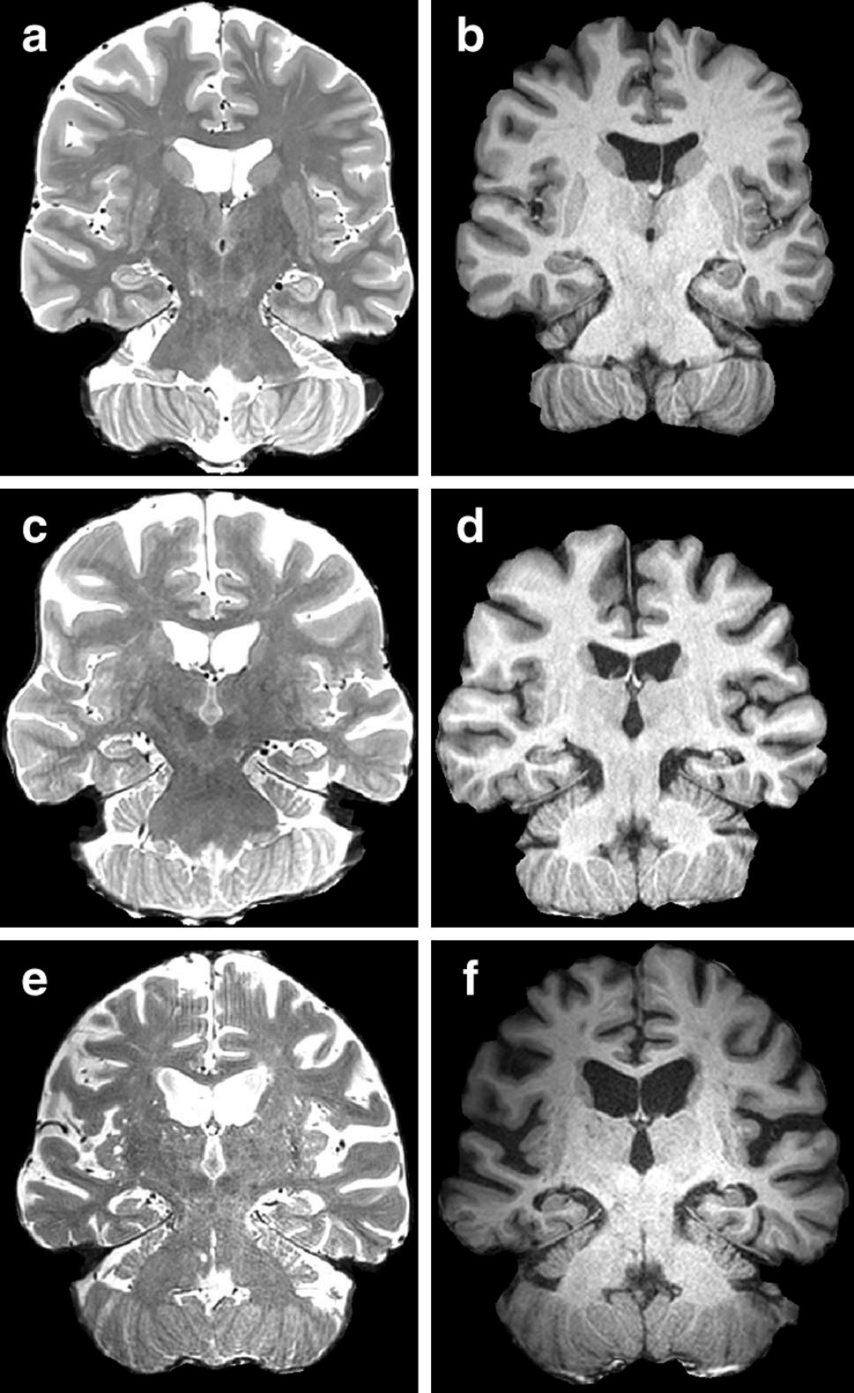
T_1_-weighted and T_2_-weighted images in individuals of different ages. *Panels* **a**, **c**, **e** show T_2_-weighted spin-echo images, *panels* **b**, **d**, **f** show T_1_-weighted MPRAGE images. *Panels* **a**, **b** are images of a 50-year-old subject, *panels* **c**, **d** a 69-year-old and **e**, **f** an 87-year-old. A decline in contrast with age between GM and WM in both types of image is visible

T_1_-weighted and T_2_-weighted images highlight the change in global image contrast that accompanies ageing (Fig. 1). A decline in general image contrast across the images, in addition to conspicuous macroscopic structural alterations, for both T_1_-weighted and T_2_-weighted images, is evident with increasing age.

**Fig. 2:**
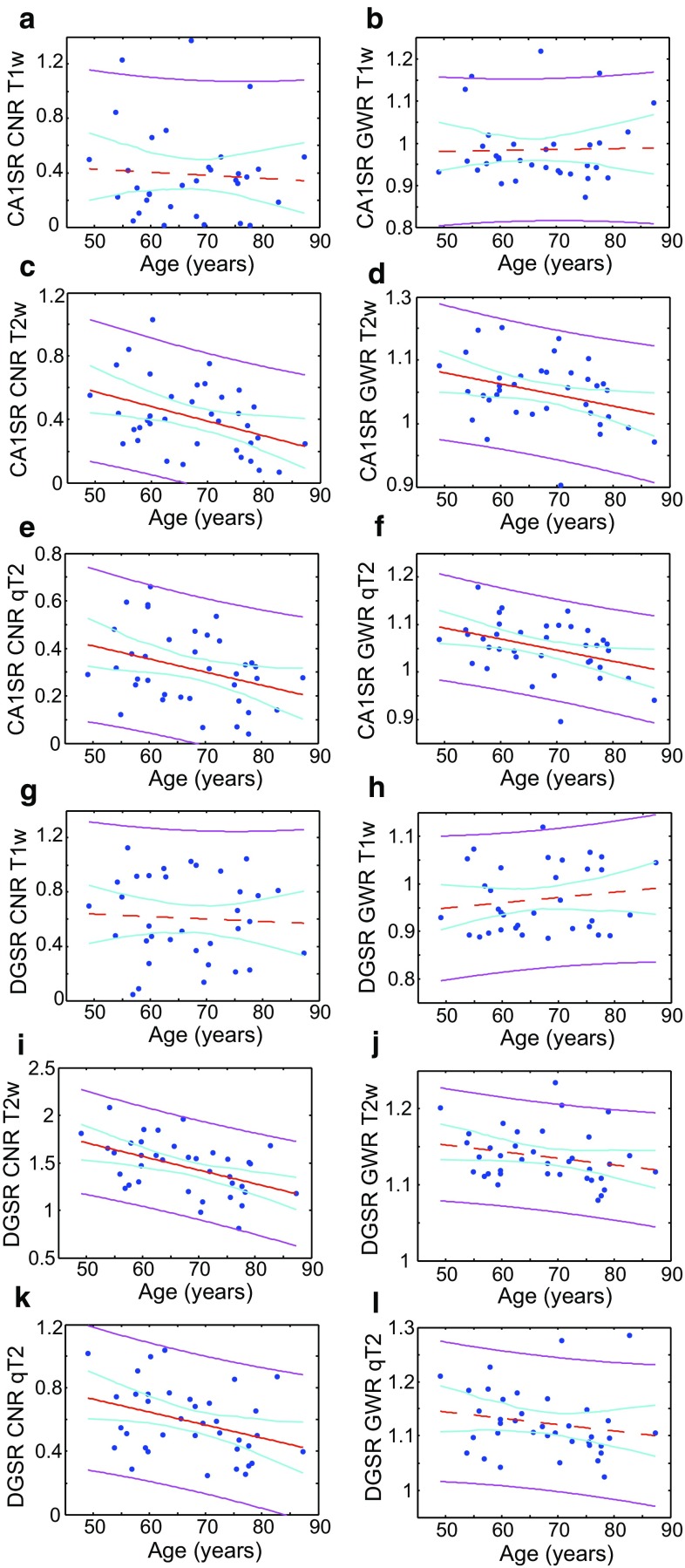
CNR and GWR in the hippocampus plotted against age. The* left panels* show CNR measurements, whilst the* right panels* show GWR measurements. The *uppermost three rows* (**a**–**f**) contrast the CA1 and SR/SL/SM subfields, whilst the *lower three rows* (**g**–**l**) contrast the DG and SR/SL/SM subfields. *Panels* **a**, **b** show CNR and GWR, respectively, for T_1_-weighted images, whilst *panels* **c**, **d** show CNR and GWR, respectively, for T_2_-weighted images, contrasting the CA1 and SL/SR/SM subfields. *Panels* **e**, **f** show CNR and GWR, respectively, for T_1_-weighted images, whilst *panels* **g**, **h** show CNR and GWR, respectively, for T_2_-weighted images, contrasting the DG and SL/SR/SM subfields. The *red line* shows a linear fit, the *magenta lines* are the 95 % confidence bounds for observations, and the *cyan lines* are the 95 % functional prediction intervals obtained by bias-corrected bootstrapping. The *red line* is solid if *p* < 0.05, and *dashed* otherwise. Fitted parameters can be found in the Supplementary Information

Figure 2 shows the age dependence of the CNR and GWR in T_1_ and T_2_-weighted images in the hippocampal body, as well as in quantitative T_2_ maps, contrasting the (GM) CA1 and DG subfields with the (WM) SL/SR/SM subfield. We observed an effect of age in the T_2_-weighted CNR and GWR, but not in T_1_-weighted images. T_2_-weighted CNR between either CA1 or DG with the SL/SR/SM subfield was also generally higher than T_1_-weighted CNR. The measurements presented in Fig. 2 may be corrupted by poor placement of hippocampal subfield boundaries, for which reason we assessed inter-rater and intra-rater reliability. For intra-rater analysis, the average measured intraclass correlation (ICC) was 0.993 with a 95 % confidence interval from 0.986 to 0.996 (*F*(62, 62) = 168.911, *p* < 0.001). For inter-rater analysis, the average measured ICC was 0.946 with a 95 % confidence interval from 0.842 to 0.982 (*F*(14, 14) = 19.150, *p* < 0.001). The Dice Kappa statistics for a subset of six hippocampi were also calculated after re-segmentation by the original rater. For those subfields used in this paper, we obtained the following Dice Kappa results. CA1: mean 0.76 with a 95 % confidence interval from 0.67 to 0.98. DG: mean 0.77 with a 95 % confidence interval from 0.58 to 0.99. SL/SR/SM: mean 0.77 with a 95 % confidence interval from 0.64 to 0.95. An additional Monte-Carlo analysis, by which means boundaries were perturbed computationally, can be found in the Supplementary Information. Measures of inter-subfield contrast were reasonably stable under perturbations to the boundaries of sizes, consistent with the discrepancies in labelling across the literature [23].

**Fig. 3:**
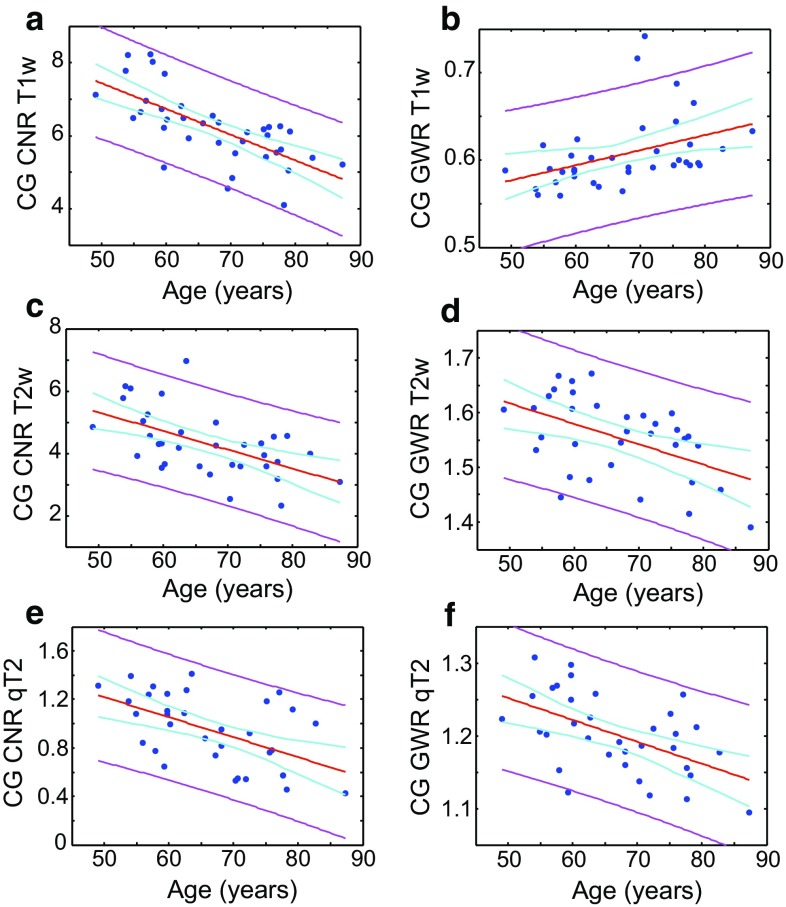
CNR and GWR in the cingulate gyrus plotted against age. The *left column* shows CNR measurements, the right displays GWR measurements. Rows are ordered in terms of T_1_-weighted images (**a**, **b**), T_2_-weighted images (**c**, **d**), and quantitative T_2_ (**e**, **f**). Specifically, *panels* **a**, **b** show CNR and GWR, respectively, for T_1_-weighted images, **c**, **d** CNR and GWR, respectively, for T_2_-weighted images. *Panels* **e**, **f** show CNR and GWR, respectively, in quantitative T_2_ maps. The *red line* shows a linear fit, the *magenta lines* are the 95 % confidence bounds for observations, and the *cyan lines* are the 95 % functional prediction intervals obtained by bias-corrected bootstrapping. The *red line* is solid if *p* < 0.05, and *dashed* otherwise. Fitted parameters can be found in the Supplementary Information

We also compared the effects of age in the CNR and GWR in T_1_-weighted and T_2_-weighted images for the cingulate gyrus (Fig. 3), a structure showing atrophy in AD [29]. Substantial effects of age are quantifiable for both contrasts. A clear normalisation of GWR towards unity is also seen for both MR contrasts with increasing age, with rather stronger correlations than in the hippocampus. The CNR and GWR obtained using quantitative T_2_ maps as a function of age are shown (Fig. 3e, f). The T_2_ GWR here is simply the ratio of the mean T_2_ between GM and WM, whereas the T_2_ CNR is the absolute difference normalised by root variance. Thus, the T_2_ in the GM of the cingulate gyrus and in the adjacent WM become more similar with age.

**Fig. 4:**
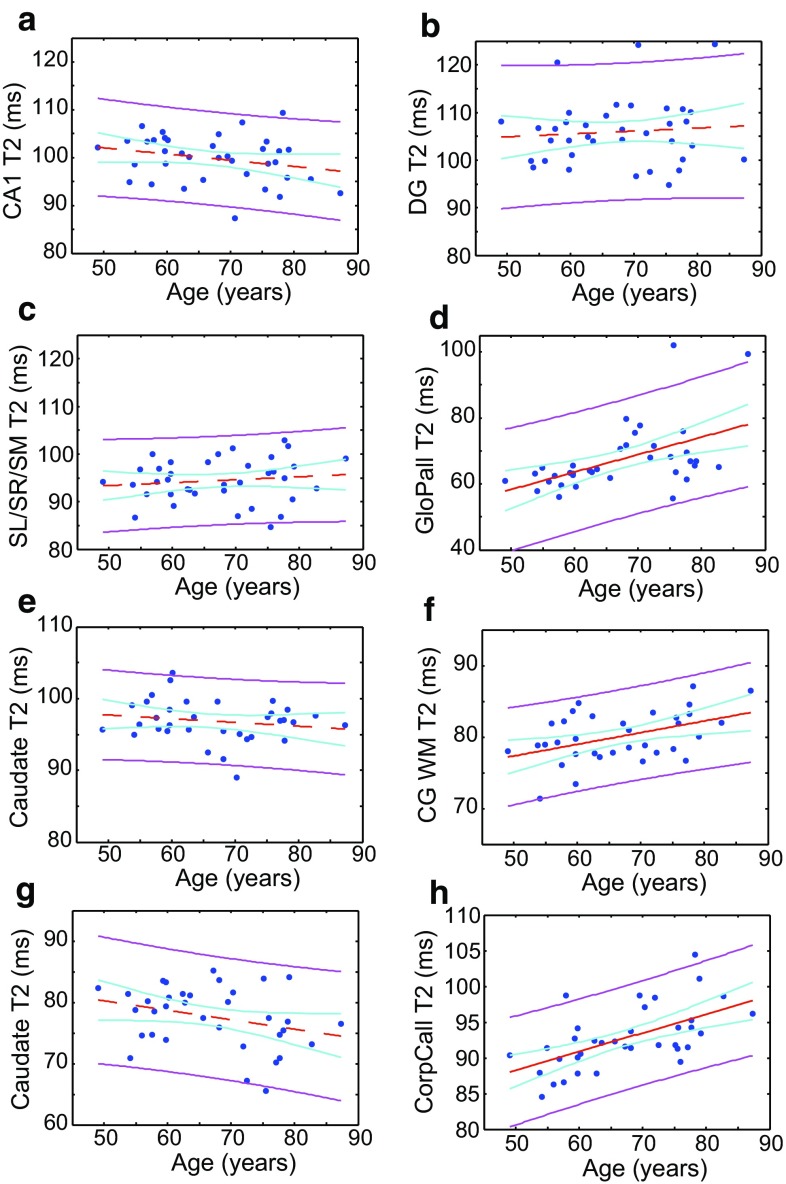
The T_2_ of various regions plotted against age. *Panels* **a**–**c** show the T_2_ of the CA1, DG, and SL/SR/SM subfields of the hippocampus, respectively. *Panel* **d** Globus pallidus, *panel* **e** cingulate (GM), *panel* **f** WM adjacent to cingulate, *panel* **g** caudate nucleus, *panel* **h** genu of corpus callosum. The *red line* shows a linear fit, the *magenta lines* are the 95 % confidence bounds for observations, and the *cyan lines* are the 95 % functional prediction intervals obtained by bias-corrected bootstrapping. The *red line* is solid if *p* < 0.05, and *dashed* otherwise. Fitted parameters can be found in the Supplementary Information

It is instructive to consider whether age-related differences in metrics of relative signal intensity, i.e., CNR and GWR, are more dependent on relaxation characteristics of one or the other tissues being contrasted. Therefore, in Fig. 4 we present the T_2_ values obtained in various regions, including caudate nucleus and globus pallidus (both belonging to deep GM) and the genu of corpus callosum (presenting myelinated WM). T_2_ in the three subfields of the hippocampus was examined (Fig. 4a–c), and no effect of age was detectable. Likewise, the cingulate T_2_ and caudate nucleus have consistent values with increasing age. However, the WM adjacent to the cingulate, as well as the genu of the corpus callosum, show substantial increases in T_2_ with age. An increase in that of the globus pallidus is also detected (all judged on the criteria *p* < 0.05 for a linear fit).

**Fig. 5:**
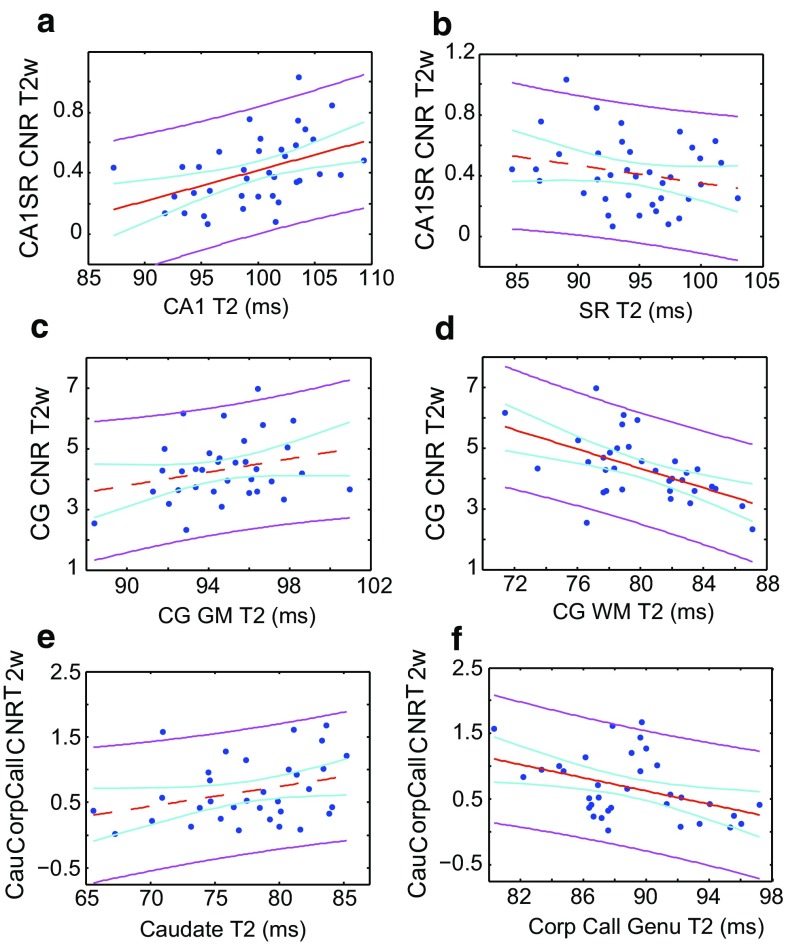
Dependence of contrast parameters on GM and WM T_2_. *Panels* **a**, **b** respectively show the dependence of T_2_-weighted contrast between the CA1 and SL/SR/SM hippocampal subfields on GM and WM T_2_. *Panels* **c**, **d** show the dependence of T_2_-weighted contrast between the cingulate gyrus and its adjacent WM on the T_2_ of the respective regions. *Panels* **e**, **f** respectively show the dependence of T_2_-weighted contrast between the caudate nucleus and corpus callosum on the T_2_ of the respective regions. The *red line* shows a linear fit, the *magenta lines* indicate the 95 % confidence bounds for observations, and the *cyan lines* are the 95 % functional prediction intervals obtained by bias-corrected bootstrapping. The *red line* is solid if p < 0.05, and *dashed* otherwise. Fitted parameters can be found in the Supplementary Information

Although the correlation between age and T_2_-weighted CNR is clear, it is not necessarily causal. To further analyse whether the GM or WM T_2_ had more of an influence on contrast between the two tissue types, we present correlations between T_2_ and T_2_-weighted CNR (Fig. 5). As representative examples, contrast between the cingulate and its adjacent WM, the hippocampal CA1 and SL/SR/SM subfields, and the caudate nucleus and corpus callosum were analysed. The general outcome is that where extensively myelinated WM is involved, contrast declines with increasing WM T_2_, with a modest contrast increase (though not significant at the 95 % level) with increasing GM T_2_. In the hippocampus, where myelination is less prevalent, a more substantive increase in contrast with increasing GM T_2_ and minimal (*p* > 0.05) negative correlation with WM T_2_ was observed.

## Discussion

### Summary of findings

As a summary of our findings, we have observed strong correlations between age and various metrics of MR image contrast in healthy elderly subjects in the cingulate gyrus and surrounding frontal WM, as well as between individual subfields of the hippocampus. This includes the use of quantitative T_2_ images and a detailed examination of individual hippocampal subfields, where quantitative T_2_ CNR and GWR also correlate with age. Ours is amongst the first studies to extend quantitative relaxometry to individual hippocampal subfields. In myelinated WM (such as the corpus callosum and the WM adjacent to the cingulate), but not the substantially less myelinated WM of the SL/SR/SM hippocampal subfield [30], T_2_ increased with age whilst GM T_2_, like non-myelinated WM, stayed constant with age.

### A possible explanation for the findings

The current data indicate cross-sectional, age-related differences for CNR and GWR between the cingulate and its adjacent WM in T_1_ and T_2_-weighted images (Fig. 3), but in the hippocampus such effects are weaker and only apparent in T_2_-weighted images (Fig. 2). This is mirrored by the observation that no age-related differences in T_2_ could be observed in the hippocampus, though a distinct value for each subfield was obtained, the (mainly WM) SL/SR/SM subfield having the shortest. On the other hand, in more extensively myelinated and macroscopically ordered WM such as the corpus callosum (and adjacent to cingulate cortex) the T_2_ increases cross-sectionally with age (Fig. 4).

We have used mono-exponential fits to our spin-echo dephasing data to obtain effective T_2_ maps. Since a single voxel of an MR image contains a dynamically mixed ensemble of spins (many different molecules interacting with one another across many timescales), we can expect that even where the criteria for mono-exponential loss of signal are met (which, on data-driven grounds, they are here), we observe a mixture of relaxation (T_2_ processes) and dynamic dephasing processes. With effective refocussing pulses, we can legitimately assume that static dephasing is negligible. With that in mind, the heuristic mono-exponential time constants used here to describe the loss of refocussable spin-phase coherence at rather long echo times (24–120 ms, spaced by 12 ms) can only somewhat colloquially be referred to as “T_2_”. We can anticipate that dynamic dephasing is a major contributor to loss of signal in a spin echo experiment (and hence, to our effective T_2_). It is driven by interactions of water with macromolecules and by diffusion of water through anisotropic magnetic fields such as those created by the myelin sheath owing to its diamagnetic susceptibility being different from its surroundings. As such, a change in myelination is likely to yield a change in effective T_2_, even at long echo times, through dynamic dephasing. This may be the cause of the age-dominated changes in myelinated WM T_2_ changes with age, as myelination is known to decrease with age [31].

The argument is supported by the existing literature demonstrating that T_1_-weighted image contrast is dominated by myelination [32–34], which we have found here also to be strongly age-dependent only where myelinated axons are involved. Likewise, T_2_-weighted imaging and quantitative T_2_ can be used as a proxy of myelin-associated water content in the brain [35, 36]. Image contrast can therefore be interpreted directly in terms of myelination. There is a high degree of order in WM, both at the molecular level where oriented layers of myelin surround axons, and at the macroscopic level where large groups of axons may maintain a common orientation in the major fiber bundles. This is not so in GM, and may also be relevant to the changes in T_2_ in (myelinated) WM as compared to conserved GM and non-myelinated WM T_2_.

Since ageing is a multi-factorial process, we must consider that many other factors are likely to play a role, but focussing on those that are likely to make the largest observable differences to MR parameters is a pragmatic and testable way to proceed.

### Relation to DTI literature

There has been considerable work on the changes of diffusion parameters in the brain with age using DTI. In particular, the fractional anisotropy (FA) of many WM tracts declines after a certain age is passed (~30) [37], whereas mean diffusivity (MD) increases with age [38]. This technique is highly sensitive to tissue microstructure and water content, changes in which are also determinants of signals in other MR modalities since most MRI approaches are determined by hydrodynamics. The decline in FA and increase in MD commensurate with age implies an increased tissue water content or degradation of anisotropic barriers to diffusion on length scales $\sqrt {{\text{MD}}t}$ where MD is mean diffusivity and *t* is the time over which diffusion is sampled (the time for which diffusion-sensitising gradients are applied). The latter mechanism implies an alteration of tissue microstructure. However, in post-mortem brains no significant change in water content was observed in subjects without neurological disorders [38]. This being so, the hydrodynamic properties of GM and WM are made more similar with increasing age, so a decline in their distinction by either T_1_-weighted or T_2_-weighted imaging, both of which are highly sensitive to hydrodynamics, is inevitable. It is easy to imagine that changes to tissue microstructure on a length scale $\sqrt {{\text{MD}}t}$ would also result in changes to dynamic dephasing. It is more difficult to imagine that tissue microstructure could be changed without altering diamagnetic susceptibilities of the various materials comprising tissues (and hence, T_2_). In this regard, the diffusion literature vindicates (and motivates) our suggestions in the preceding section: that T_2_ increases in WM due to the degradation of ordered structures, of which the myelin sheath is a prime, though not unique, candidate.

### Relation to other qMRI research

Work combining MT and quantitative T_1_ and T_2_* mapping has also shown that MT decreases with age (on a cross-sectional basis) in various GM structures, but not in the hippocampus or cingulate gyrus [4]. T_1_ was also reported to be less sensitive to age than the other MR parameters used. Similar findings have also been reported in a study extending such methods to a larger cohort and employing statistical parametric mapping [3]. In both the latter studies [3, 4], T_2_* was used, in distinction to ours, which uses spin-echo T_2_ and is less affected by static dephasing. Thus, the information content of the two parameters is rather different. Recent work has shown that T_2_* is highly sensitive to iron content in deep GM structures, significantly more so than T_2_, and that there exist different age effects on T_2_ and T_2_* for different GM structures and WM regions [39]. Our observation of a modest increase of the T_2_ in the globus pallidus with age is in agreement with the latter study, though ours is a somewhat different cohort. The former study demonstrates a substantial decrease in T_2_* in the iron-rich globus pallidus with increasing age. The increased T_2_ we have found with age in iron-rich regions is unlikely to be reflective of iron accumulation but rather of accompanying changes that decrease the dynamic dephasing contributions to decoherence of the nuclear spin phase.

Arterial spin labelling (ASL) has also revealed age-related changes in cross-sectional studies of cerebral perfusion [40, 41]. We may therefore speculate that given cerebral perfusion changes with age, and that by virtue of alterations in the amount of (paramagnetic) deoxyhaemoglobin present in the capillary bed and venules, effective T_2_ may be perturbed by such a mechanism (the capillary bed will cause decoherence by creating an inhomogeneous local field). However, cerebral perfusion changes to a greater extent in GM than WM, whereas our age-related T_2_ changes were larger in WM than GM. Whilst such a mechanism is certainly relevant to ageing, it may be less relevant to changes in T_2_, and to T_1_-weighted CNR and GWR.

### Relation to volumetry

It is well appreciated that the hippocampus and cingulate suffer atrophy in MCI and AD [42]. Furthermore, atrophy rates in the hippocampus [43–45] and cingulate [29, 46] are able to distinguish between healthy ageing, MCI and AD, but an understanding of the ageing process beyond volume changes may be pertinent to dementia research and clinical diagnosis. The distinct functions of the different hippocampal subfields, combined with the progression of AD pathology through those subfields, suggests that our approach to quantifying tissue characteristics, such as CNR and GWR, at an individual subfield level may in the future be useful in detecting early dementia risk or distinguishing between different dementia pathologies. This adds to an expanding literature examining the hippocampus by MRI at the subfield level, rather than considering its entire volume [23, 47–49]. Recent data point to localised changes in WM of the hippocampus in the SL/SR/SM subfield group, analysed here in a tauopathy mouse model for AD [50]. This accelerates the need for a detailed characterisation of healthy hippocampal WM aging at the level of individual subfields, and the identification of the MRI parameters sensitive to deviations from such a pathway at early stages.

### Methodological challenges and limitations

When using CNR, as stated in the methods section, it is important to ensure that the variance in the regions of interest derives from sources of random (stochastic) noise, and not from slowly varying (deterministic) magnetic field inhomogeneities, manifest as gradual shading across regions of an image, hence the use of receiver coil sensitivity corrections (‘prescan normalize’ on the particular scanner used) and bias field corrections. This is far less an issue when using quantitative T_2_ maps, though to some extent is still present (in particular due to transmitter B_1_ inhomogeneity, which causes RF-pulse flip angle variation leading to incomplete refocussing). Obtaining meaningful results for CNR estimates of image intensities therefore requires the use of complementary ROIs that are close in image space and over which “shading” is irrelevant, but which nevertheless sample sufficient voxels to determine the mean and variance with high precision. This is in contrast to volume determination, where an ROI must fully represent the structure of interest, irrespective of image intensity. CNR and GWR estimates have the advantage, however, that ROIs need only sample sufficient voxels to be representative, rather than capturing the shape and thus volume of a structure. Provided the first conditions of this section are met, the result is that substantial freedom in ROI preparation is permissible whilst still generating a stable CNR or GWR estimate that would not be acceptable if attempting volume determination.

Our careful approach to data analysis with conservative ROIs and use of high-performance phase-arrayed coils, as well as the B_0_ field strength of 3 T in concert is believed to explain the discrepancy between our findings and earlier studies; the work of Magnaldi et al. [24] reported a negative correlation between WM T_2_ and age, which is rather contradicted here. Work by Kim et al. to determine GWR in T_2_-weighted images using ROIs covering all subcortical structures (and thus not obeying the conditions for GWR representative of physiology rather than instrumental performance) obtained considerably smaller effects than reported here [25].

Our study, like others using different qMRI parameters [3, 9, 51], also suffers from the limitation of being cross-sectional rather than longitudinal, though in covering an age range of 38 years this may have been difficult to overcome practically. The consequence is that factors other than age may spuriously contribute to our findings. The future extension to a larger cohort or a longitudinal study over a respectable duration may ameliorate these limitations.

## Conclusions

Overall, it is likely that several mechanisms are simultaneously responsible for changes in MR contrast, present to different extents in different regions of the brain that need to be considered in design of volumetric protocols in aged brain. Tissue contrast can and should be interpreted directly in terms of microscopic and/or physico-chemical aspects of the tissues that comprise the systems being imaged, with quantitative imaging providing a further window into their properties. We anticipate the detailed examination presented here as having applications in dementia imaging research, as well as contributing to an understanding of healthy aging.

## Electronic supplementary material

Below is the link to the electronic supplementary material.

Supplementary material 1 (DOCX 286 kb)

## References

[1] Leenders KL, Perani D, Lammertsma AA, Heather JD, Buckingham P, Healy MJ, Gibbs JM, Wise RJ, Hatazawa J, Herold S (1990). Cerebral blood flow, blood volume and oxygen utilization. Normal values and effect of age. Brain.

[2] Tang Y, Nyengaard JR, Pakkenberg B, Gundersen HJ (1997). Age-induced white matter changes in the human brain: a stereological investigation. Neurobiol Aging.

[3] Callaghan MF, Freund P, Draganski B, Anderson E, Cappelletti M, Chowdhury R, Diedrichsen J, Fitzgerald TH, Smittenaar P, Helms G, Lutti A, Weiskopf N (2014). Widespread age-related differences in the human brain microstructure revealed by quantitative magnetic resonance imaging. Neurobiol Aging.

[4] Draganski B, Ashburner J, Hutton C, Kherif F, Frackowiak RS, Helms G, Weiskopf N (2011). Regional specificity of MRI contrast parameter changes in normal ageing revealed by voxel-based quantification (VBQ). Neuroimage.

[5] Chang L, Ernst T, Poland RE, Jenden DJ (1996). In vivo proton magnetic resonance spectroscopy of the normal aging human brain. Life Sci.

[6] House MJ, St Pierre TG, Kowdley KV, Montine T, Connor J, Beard J, Berger J, Siddaiah N, Shankland E, Jin LW (2007). Correlation of proton transverse relaxation rates (R2) with iron concentrations in postmortem brain tissue from Alzheimer’s disease patients. Magn Reson Med.

[7] Ogg RJ, Steen RG (1998). Age-related changes in brain T1 are correlated with iron concentration. Magn Reson Med.

[8] Jack CR (2012). Alzheimer disease: new concepts on its neurobiology and the clinical role imaging will play. Radiology.

[9] Salat DH, Lee SY, van der Kouwe AJ, Greve DN, Fischl B, Rosas HD (2009). Age-associated alterations in cortical gray and white matter signal intensity and gray to white matter contrast. Neuroimage.

[10] Scahill RI, Fox NC (2007). Longitudinal imaging in dementia. Br J Radiol.

[11] Frisoni GB, Fox NC, Jack CR, Scheltens P, Thompson PM (2010). The clinical use of structural MRI in Alzheimer disease. Nat Rev Neurol.

[12] Fox NC, Schott JM (2004). Imaging cerebral atrophy: normal ageing to Alzheimer’s disease. Lancet.

[13] McDonald CR, McEvoy LK, Gharapetian L, Fennema-Notestine C, Hagler DJ, Holland D, Koyama A, Brewer JB, Dale AM, For the Alzheimer’s Disease Neuroimaging I (2009). Regional rates of neocortical atrophy from normal aging to early Alzheimer disease. Neurology.

[14] Whitwell JL (2010). Progression of atrophy in Alzheimer’s disease and related disorders. Neurotox Res.

[15] Whitwell JL, Przybelski SA, Weigand SD, Knopman DS, Boeve BF, Petersen RC, Jack CR (2007). 3D maps from multiple MRI illustrate changing atrophy patterns as subjects progress from mild cognitive impairment to Alzheimer’s disease. Brain.

[16] Li J, Pan P, Huang R, Shang H (2012). A meta-analysis of voxel-based morphometry studies of white matter volume alterations in Alzheimer’s disease. Neurosci Biobehav Rev.

[17] Wang WY, Yu JT, Liu Y, Yin RH, Wang HF, Wang J, Tan L, Radua J, Tan L (2015). Voxel-based meta-analysis of grey matter changes in Alzheimer’s disease. Transl Neurodegener.

[18] Pan PL, Song W, Yang J, Huang R, Chen K, Gong QY, Zhong JG, Shi HC, Shang HF (2012). Gray matter atrophy in behavioral variant frontotemporal dementia: a meta-analysis of voxel-based morphometry studies. Dement Geriatr Cogn Disord.

[19] Pan PL, Shi HC, Zhong JG, Xiao PR, Shen Y, Wu LJ, Song YY, He GX, Li HL (2013). Gray matter atrophy in Parkinson’s disease with dementia: evidence from meta-analysis of voxel-based morphometry studies. Neurol Sci.

[20] Shi HC, Zhong JG, Pan PL, Xiao PR, Shen Y, Wu LJ, Li HL, Song YY, He GX, Li HY (2013). Gray matter atrophy in progressive supranuclear palsy: meta-analysis of voxel-based morphometry studies. Neurol Sci.

[21] Westlye LT, Walhovd KB, Dale AM, Espeseth T, Reinvang I, Raz N, Agartz I, Greve DN, Fischl B, Fjell AM (2009). Increased sensitivity to effects of normal aging and Alzheimer’s disease on cortical thickness by adjustment for local variability in gray/white contrast: a multi-sample MRI study. Neuroimage.

[22] Salat DH, Chen JJ, van der Kouwe AJ, Greve DN, Fischl B, Rosas HD (2011). Hippocampal degeneration is associated with temporal and limbic gray matter/white matter tissue contrast in Alzheimer’s disease. Neuroimage.

[23] Yushkevich PA, Amaral RS, Augustinack JC, Bender AR, Bernstein JD, Boccardi M, Bocchetta M, Burggren AC, Carr VA, Chakravarty MM, Chetelat G, Daugherty AM, Davachi L, Ding SL, Ekstrom A, Geerlings MI, Hassan A, Huang Y, Iglesias JE, La Joie R, Kerchner GA, LaRocque KF, Libby LA, Malykhin N, Mueller SG, Olsen RK, Palombo DJ, Parekh MB, Pluta JB, Preston AR, Pruessner JC, Ranganath C, Raz N, Schlichting ML, Schoemaker D, Singh S, Stark CE, Suthana N, Tompary A, Turowski MM, Van Leemput K, Wagner AD, Wang L, Winterburn JL, Wisse LE, Yassa MA, Zeineh MM (2015). Quantitative comparison of 21 protocols for labeling hippocampal subfields and parahippocampal subregions in in vivo MRI: towards a harmonized segmentation protocol. Neuroimage.

[24] Magnaldi S, Ukmar M, Vasciaveo A, Longo R, Pozzi-Mucelli RS (1993). Contrast between white and grey matter: MRI appearance with ageing. Eur Radiol.

[25] Kim DM, Xanthakos SA, Tupler LA, Barboriak DP, Charles HC, MacFall JR, Krishnan KR (2002). MR signal intensity of gray matter/white matter contrast and intracranial fat: effects of age and sex. Psychiatry Res.

[26] Zhang Y, Brady M, Smith S (2001). Segmentation of brain MR images through a hidden Markov random field model and the expectation-maximization algorithm. IEEE Trans Med Imaging.

[27] Wood B, Knight MJ, Tsivos D, Oliver R, Coulthard E, Kauppinen RA (2015). Magnetic resonance scanning and image segmentation procedure at 3 T for volumetry of human hippocampal subfields. Biomed Spectrosc Imaging.

[28] Mueller SG, Schuff N, Yaffe K, Madison C, Miller B, Weiner MW (2010). Hippocampal atrophy patterns in mild cognitive impairment and Alzheimer’s disease. Hum Brain Mapp.

[29] Barnes J, Godbolt AK, Frost C, Boyes RG, Jones BF, Scahill RI, Rossor MN, Fox NC (2007). Atrophy rates of the cingulate gyrus and hippocampus in AD and FTLD. Neurobiol Aging.

[30] Andersen P, Morris R, Amaral D, Bliss T, O’Keefe J (2006). The hippocampus book.

[31] Peters A (2002). The effects of normal aging on myelin and nerve fibers: a review. J Neurocytol.

[32] Bock NA, Kocharyan A, Liu JV, Silva AC (2009). Visualizing the entire cortical myelination pattern in marmosets with magnetic resonance imaging. J Neurosci Methods.

[33] Glasser MF, Goyal MS, Preuss TM, Raichle ME, Van Essen DC (2014). Trends and properties of human cerebral cortex: correlations with cortical myelin content. Neuroimage.

[34] Lutti A, Dick F, Sereno MI, Weiskopf N (2014). Using high-resolution quantitative mapping of R1 as an index of cortical myelination. Neuroimage.

[35] Alonso-Ortiz E, Levesque IR, Pike GB (2015). MRI-based myelin water imaging: a technical review. Magn Reson Med.

[36] Wilhelm MJ, Ong HH, Wehrli SL, Li C, Tsai P-H, Hackney DB, Wehrli FW (2012). Direct magnetic resonance detection of myelin and prospects for quantitative imaging of myelin density. Proc Natl Acad Sci USA.

[37] Kochunov P, Williamson DE, Lancaster J, Fox P, Cornell J, Blangero J, Glahn DC (2012). Fractional anisotropy of water diffusion in cerebral white matter across the lifespan. Neurobiol Aging.

[38] Besson JA, Best PV, Skinner ER (1992). Post-mortem proton magnetic resonance spectrometric measures of brain regions in patients with a pathological diagnosis of Alzheimer’s disease and multi-infarct dementia. Br J Psychiatry.

[39] Sedlacik J, Boelmans K, Lobel U, Holst B, Siemonsen S, Fiehler J (2014). Reversible, irreversible and effective transverse relaxation rates in normal aging brain at 3T. Neuroimage.

[40] Biagi L, Abbruzzese A, Bianchi MC, Alsop DC, Del Guerra A, Tosetti M (2007). Age dependence of cerebral perfusion assessed by magnetic resonance continuous arterial spin labeling. J Magn Reson Imaging.

[41] Liu Y, Zhu X, Feinberg D, Guenther M, Gregori J, Weiner MW, Schuff N (2012). Arterial spin labeling MRI study of age and gender effects on brain perfusion hemodynamics. Magn Reson Med.

[42] McConathy J, Sheline YI (2015). Imaging biomarkers associated with cognitive decline: a review. Biol Psychiatry.

[43] Barnes J, Bartlett JW, van de Pol LA, Loy CT, Scahill RI, Frost C, Thompson P, Fox NC (2009). A meta-analysis of hippocampal atrophy rates in Alzheimer’s disease. Neurobiol Aging.

[44] Hänggi J, Streffer J, Jäncke L, Hock C (2011). Volumes of lateral temporal and parietal structures distinguish between healthy aging, mild cognitive impairment, and Alzheimer’s disease. J Alzheimers Dis.

[45] Leung KK, Bartlett JW, Barnes J, Manning EN, Ourselin S, Fox NC (2013). Cerebral atrophy in mild cognitive impairment and Alzheimer disease: rates and acceleration. Neurology.

[46] Pengas G, Hodges JR, Watson P, Nestor PJ (2010). Focal posterior cingulate atrophy in incipient Alzheimer’s disease. Neurobiol Aging.

[47] Winterburn JL, Pruessner JC, Chavez S, Schira MM, Lobaugh NJ, Voineskos AN, Chakravarty MM (2013). A novel in vivo atlas of human hippocampal subfields using high-resolution 3 T magnetic resonance imaging. Neuroimage.

[48] Wisse LE, Gerritsen L, Zwanenburg JJ, Kuijf HJ, Luijten PR, Biessels GJ, Geerlings MI (2012). Subfields of the hippocampal formation at 7 T MRI: in vivo volumetric assessment. Neuroimage.

[49] La Joie R, Fouquet M, Mezenge F, Landeau B, Villain N, Mevel K, Pelerin A, Eustache F, Desgranges B, Chetelat G (2010). Differential effect of age on hippocampal subfields assessed using a new high-resolution 3T MR sequence. Neuroimage.

[50] Maurin H, Chong SA, Kraev I, Davies H, Kremer A, Seymour CM, Lechat B, Jaworski T, Borghgraef P, Devijver H, Callewaert G, Stewart MG, Van Leuven F (2014). Early structural and functional defects in synapses and myelinated axons in stratum lacunosum moleculare in two preclinical models for tauopathy. PLoS ONE.

[51] Salat DH, Tuch DS, van der Kouwe AJ, Greve DN, Pappu V, Lee SY, Hevelone ND, Zaleta AK, Growdon JH, Corkin S, Fischl B, Rosas HD (2010). White matter pathology isolates the hippocampal formation in Alzheimer’s disease. Neurobiol Aging.

