# Supplementary material for: Quantitative T1 and T2 MRI signal characteristics in the human brain: different patterns of MR contrasts in normal ageing
Source: MAGMA. 2016 Jun 22;29(6):833–42. doi: 10.1007/s10334-016-0573-0 (PMC5124042; doi:10.1007/s10334-016-0573-0)
Supplement: Supplementary file 1 — Supplementary material 1 (DOCX 286 kb) [file 10334_2016_573_MOESM1_ESM.docx]

**Supplementary Information**

**Quantitative T_1_ and T_2_ MRI signal characteristics in the human brain: different patterns of MR contrasts in normal ageing**

Michael J. Knight, Bryony Wood, Demitra Tsivos, Elizabeth Couthard and Risto A. Kauppinen


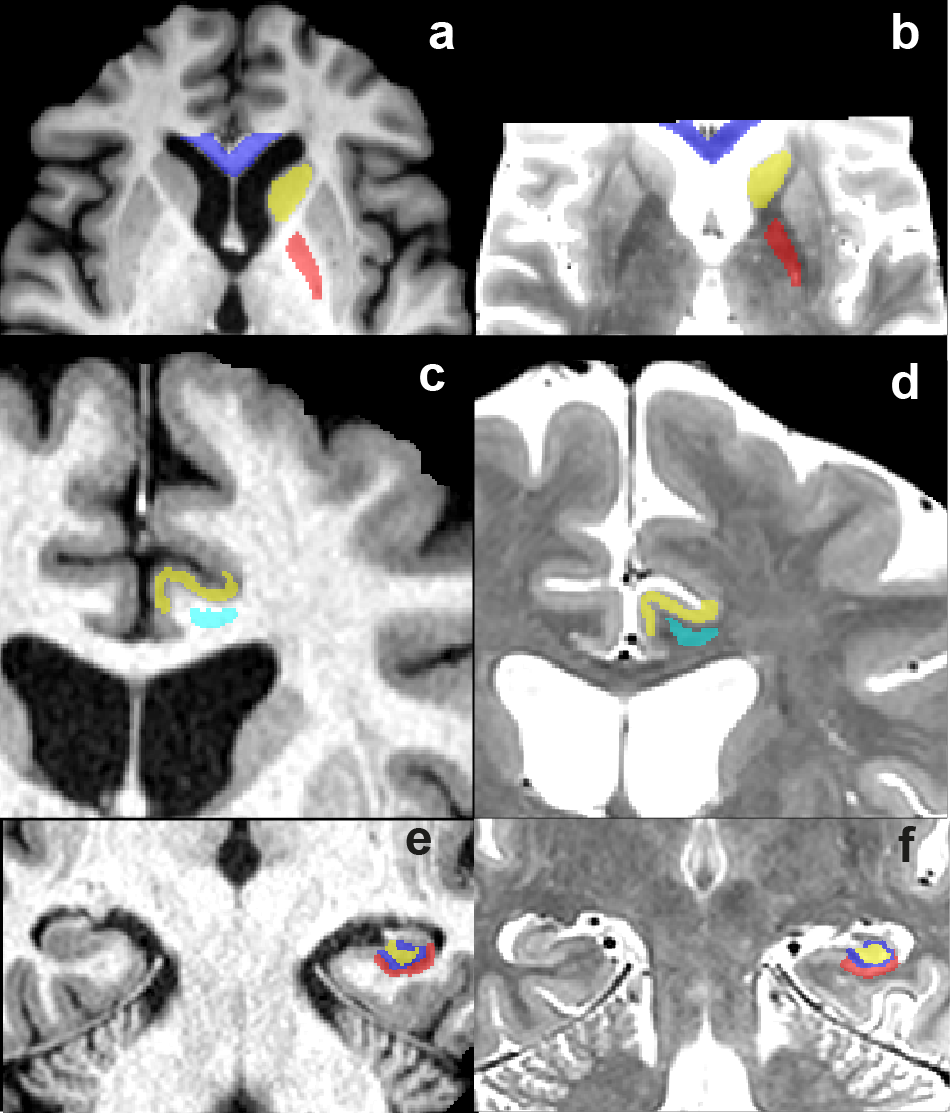


**Figure S1**: Masks used in this paper on images of a representative brain. Panels a,c,e show T_1_-weighted images, panels b,d,f show T_2_-weighted images. Panels a and b show masks of the genu of the corpus callosum (blue), caudate nucleus (yellow) and globus pallidus (red). Panels c and d show masks of the cingulate gyrus (yellow) and adjacent white matter (cyan). Panels e and f show masks of the hippocampal CA1 (red), SL/SR/SM (blue) and DG (yellow). Since masks do not need to represent shape or volume, but rather a representative set of voxels within a structure, mask boundaries are preferentially placed conservatively inside a structure boundary to fulfil the criteria of CNR and GWR calculation.

**Analysis of hippocampal CNR numerical stability**

To determine to what extent variability in the placement of hippocampal subfield boundaries may impact on measures of contrast between the selected subfields, we implemented a Monte-Carlo procedure for the alteration of subfield boundaries. This procedure was designed to randomly alter a selected boundary between two subfields (leaving all other boundaries unaltered) and re-calculate the measures of signal intensity explained below. By such performing 5000 such simulations, a distribution of values could be obtained, emulating variability in manual placement of subfield boundaries. The program was able to move boundaries by two voxels in any direction, giving a domain of uncertainty (boundary resampling domain) typically four voxels wide. Simulations were performed for the CA1- SL/SR/SM (SR in Figure 2S) boundary, the DG- SL/SR/SM boundary and additionally the CA1-SUB (subiculum) boundary. The latter was included, despite not being a main objective of the current work, as it is acknowledged as being particularly discrepant amongst hippocampal labelling protocols and thus impacts on measurements involving the CA1 subfield. The procedure was implemented for T_2_-weighted images. We found that the distribution of contrast-to-noise ratios (CNR) and grey-to-white matter ratios (GWR) had well-defined distributions. In other words, large discrepancies in boundary placement cause CNR or GWR to deliver outlying values, but they are numerically stable across quite a broad range of boundary placements. Results are shown in Figure S2.


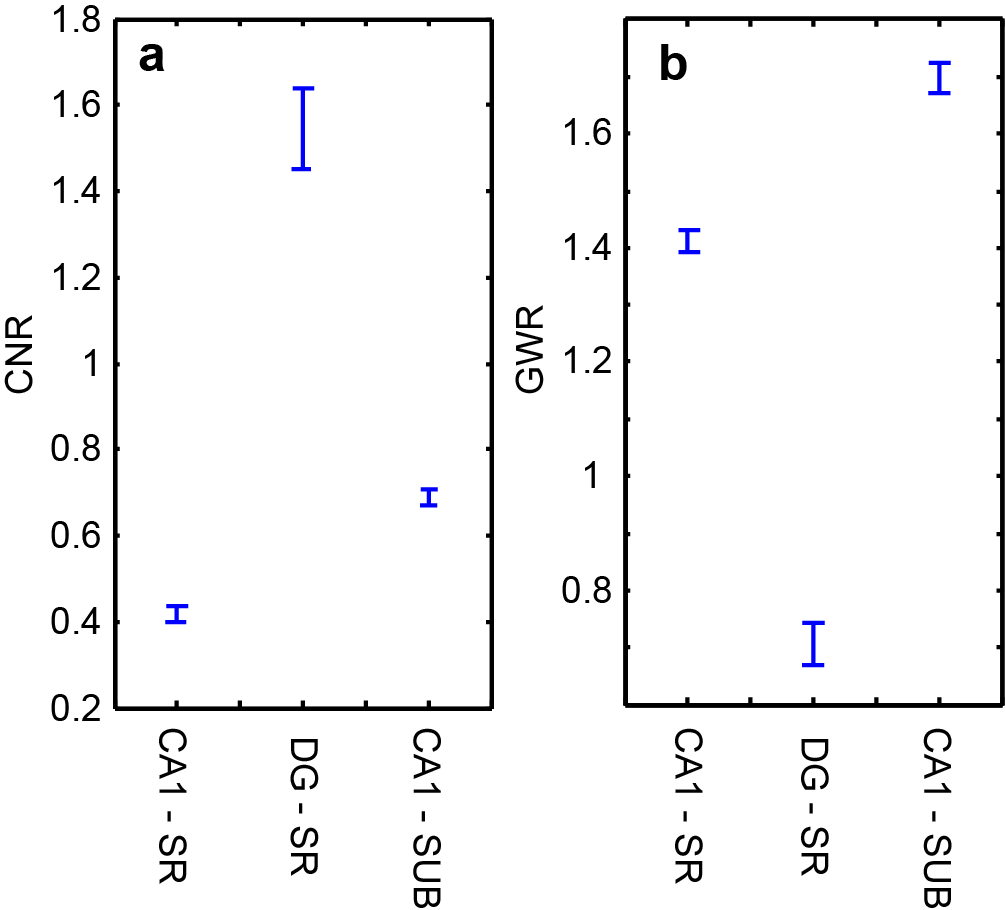


Figure S2: Stability estimates for CNR (a) and GWR (b) in the hippocampus. The figure pertains to CNR and GWR estimates using T_2_-weighted images. Bars show the width of the distribution of CNR or GWR obtained, the bar full-width being two standard deviations. The distributions of possible CNR and GWR values subject to different boundaries were assembled as described in the preceding text.

**Table S1**: Statistics pertaining to linear fits performed for the data presented in the main text. All fits were of the form Par2 = C1*Par1 + C2. Par1 and Par2 are the independent and dependent variables respectively. Units of fitted parameters and their errors may be inferred from those indicated in the Par1 and Par2 columns. Parameter uncertainties were estimated by bias-corrected bootstrapping as described in the main text. R is the Pearson correlation coefficient and Pval the p statistic. The Figure column refers to the figure panel in the main text in which the data are presented. The table is presented alphabetically, ordered by Par1, then Par2.

| **Par1** | **Par2** | **R** | **P value** | **C1** | **C2** | **Figure** |
| --- | --- | --- | --- | --- | --- | --- |
| Age (years) | CA1 T2 (ms) | -0.2588 | 0.1219 | -0.1307 ± 0.1685 | 108.6000 ± 10.6 | 4a |
| Age (years) | CA1SR CNR T2w | -0.3973 | 0.0149 | -0.0093 ± 0.006319 | 1.0392 ± 0.4696 | 2c |
| Age (years) | CA1SR GWR T2w | -0.3239 | 0.0505 | -0.0018 ± 0.1553 | 1.1678 ± 0.1141 | 2d |
| Age (years) | CA1SR CNR qT2 | -0.3356 | 0.0423 | -0.0055 ± 0.004951 | 0.6869 ± 0.3237 | 2e |
| Age (years) | CA1SR GWR qT2 | -0.4001 | 0.0141 | -0.0023 ± 0.001643 | 1.2095 ± 0.1427 | 2f |
| Age (years) | CA1SR CNR T1w | -0.0657 | 0.7121 | -0.0023 ± 0.01057 | 0.5412 ± 0.7475 | 2a |
| Age (years) | CA1SR GWR T1w | 0.0256 | 0.8859 | 0.0002 ± 0.002470 | 0.9708 ± 0.1765 | 2b |
| Age (years) | CGGM T2 (ms) | -0.2219 | 0.2072 | -0.0577 ± 0.1015 | 98.6688 ± 6.8724 | 4e |
| Age (years) | CG CNR qT2 | -0.5449 | 0.0009 | -0.0165 ± 0.008413 | 2.0452 ± 0.5932 | 3e |
| Age (years) | CG GWR qT2 | -0.5335 | 0.0012 | -0.0030 ± 0.001620 | 1.4025 ± 0.09879 | 3f |
| Age (years) | CGWM T2 (ms) | 0.4508 | 0.0075 | 0.1632 ± 0.1160 | 69.1816 ± 8.3431 | 4f |
| Age (years) | CG CNR T2w | -0.5606 | 0.0006 | -0.0601 ± 0.03162 | 8.3190 ± 2.0150 | 3c |
| Age (years) | CG CNR T2w | -0.7078 | 0.0000 | -0.0736 ± 0.002267 | 10.0673 ± 1.8254 | 3a |
| Age (years) | CG GWR T2w | -0.4936 | 0.0030 | -0.0037 ± 0.002435 | 1.8039 ± 0.1545 | 3d |
| Age (years) | CG GWR T1w | 0.6382 | 0.0000 | 0.0024 ± 0.001177 | 0.4381 ± 0.07358 | 3b |
| Age (years) | DG T2 (ms) | 0.0848 | 0.6179 | 0.0612 ± 0.2387 | 101.8000 ± 15.7456 | 4b |
| Age (years) | DGSR CNR T2w | -0.4818 | 0.0025 | -0.0144 ± 0.009395 | 2.4277 ± 0.4877 | 2i |
| Age (years) | DGSR GWR T2w | -0.2397 | 0.1530 | -0.0009 ± 0.001202 | 1.1963 ± 0.07578 | 2j |
| Age (years) | DGSR CNR qT2 | -0.3516 | 0.0328 | -0.0082 ± 0.006988 | 1.1383 ± 0.4436 | 2k |
| Age (years) | DGSR GWR qT2 | -0.1861 | 0.2702 | -0.0012 ± 0.002055 | 1.2028 ± 0.1197 | 2l |
| Age (years) | DGSR CNR T1w | -0.0557 | 0.7545 | -0.0018 ± 0.009568 | 0.7280 ± 0.7742 | 2g |
| Age (years) | DGSR GWR T1w | 0.1505 | 0.3956 | 0.0011 ± 0.002314 | 0.8939 ± 0.1606 | 2h |
| Age (years) | GloPall T2 (ms) | 0.5140 | 0.0013 | 0.5295 ± 0.1126 | 31.8028 ± 18.1504 | 4d |
| Age (years) | SLSRSMLR T2 (ms) | 0.1305 | 0.4415 | 0.0614 ± 0.1500 | 90.3100 ± 10.0612 | 4c |
| CA1 T2 (ms) | CA1SR CNR T2w | 0.4402 | 0.0064 | 0.0204 ± 0.01359 | -1.6170 ± 1.4093 | 5a |
| SLSRSM T2 (ms) | CA1SR CNR T2w | -0.2362 | 0.1593 | -0.0118 ± 0.01611 | 1.5220 ± 1.5014 | 5b |
| CGWM T2 (ms) | CG CNR T2w | -0.5392 | 0.0010 | -0.1596 ± 0.08087 | 17.0900 ± 6.1198 | 5d |
| CGGM T2 (ms) | CG CNR T2w | 0.2664 | 0.1278 | 0.1098 ± 0.1218 | -6.0990 ± 11.4559 | 5c |
| Caudate T2 (ms) | Cau_CorpCall CNR T2w | 0.3148 | 0.0698 | 0.0303 ± 0.02964 | -1.6780 ± 2.4842 | 5e |
| CorpCall T2 (ms) | Cau_CorpCall CNR T2w | -0.4418 | 0.0089 | -0.0489 ± 0.03806 | 5.2020 ± 3.5504 | 5f |
